# The *Schizosaccharomyces pombe* nucleolar protein Nsk1 modulates rDNA silencing during interphase

**DOI:** 10.17912/micropub.biology.001616

**Published:** 2025-05-08

**Authors:** Jun-Song Chen, Sarah M. Hanna, Alaina H. Willet, Kathleen L. Gould

**Affiliations:** 1 Department of Cell and Developmental Biology, Vanderbilt University School of Medicine, Nashville, TN, US

## Abstract

*Schizosaccharomyces pombe* Nsk1 acts at kinetochores during mitosis to prevent error-prone chromosome segregation and it is phosphoregulated by Cdk1 . The Clp1 / Cdc14 protein phosphatase, to which Nsk1 binds, reverses Cdk1-mediated phosphorylation of Nsk1 during anaphase. During interphase, Nsk1 localizes exclusively to the nucleolus and its function there is unknown. In this study, we examined whether Nsk1 shares functions in the nucleolus with other known Clp1 / Cdc14 phosphatase substrates that localize there. We found that Nsk1 participates in rRNA silencing but not rDNA segregation, rDNA transcription, or nucleolar organization.

**Figure 1. Characterization of Nsk1 nucleolar functions:**
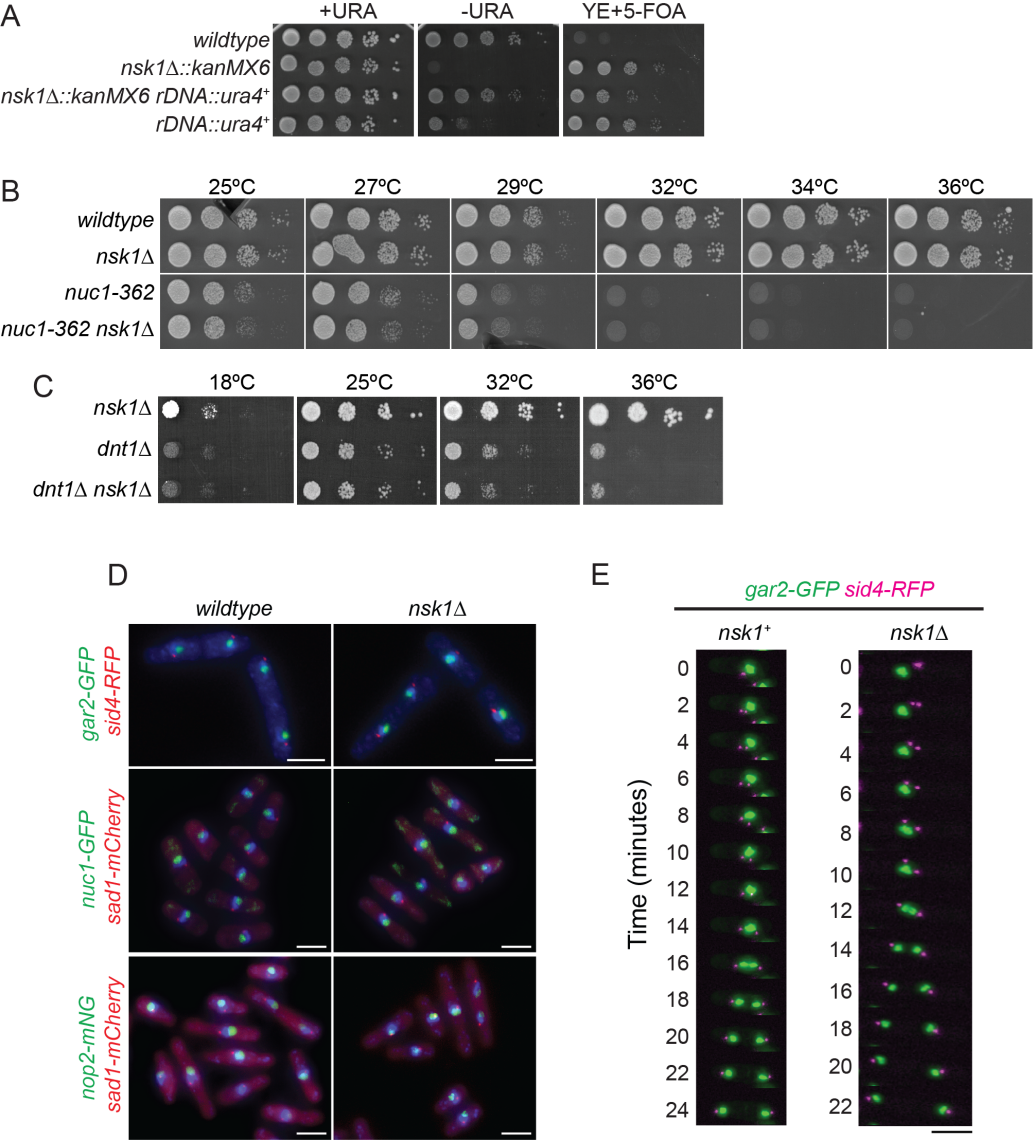
(A-C) The indicated strains were grown in liquid YE at 25°C until they reached mid-log phase and adjusted to the same cell concentrations measured by optical density (Forsburg and Rhind, 2006). Then, 10-fold serial dilutions were made and 2.5 µL of each was spotted on (A) EMM with uracil, EMM without uracil, YE with 5-FOA agar, or (B-C) YE plates and incubated at the indicated temperatures for 2-5 days prior to imaging. (D) Representative images of fixed cells of the indicated genotypes stained with DAPI to visualize DNA. Scale bars, 5 µm. (E) Representative montages of live-cell imaging of the indicated strains grown at 25˚C in YE. Time 0 is the image before SPB separation. Scale bar, 5 µm.

## Description

Previously, we and others identified *Schizosaccharomyces pombe* Nsk1 as a substrate and binding partner of the Clp1 protein phosphatase (Buttrick et al., 2011; Chen et al., 2013; Chen et al., 2011). Although *clp1 ^+^* is not essential, Clp1 dephosphorylates Cdk1 substrates and therefore plays a role in mitotic exit (reviewed in Clifford et al., 2008; Mocciaro and Schiebel, 2010; Stegmeier and Amon, 2004; Trautmann and McCollum, 2002). Nsk1 was found to localize to the kinetochore during mitosis where it acts to ensure accurate chromosome segregation and during interphase, Nsk1 localizes to the nucleolus (Buttrick et al., 2011; Chen et al., 2011).

Here, we examined whether Nsk1 plays similar roles in the nucleolus to other *S. pombe* Clp1 and *Saccharomyces cerevisiae* Cdc14 binding partners that reside in the nucleolus during interphase. In *S. cerevisiae* , Net1 is a nucleolar protein that binds and anchors Cdc14 there during interphase (Shou et al., 1999; Traverso et al., 2001; Visintin et al., 1999). *S. cerevisiae* Tof2 is a second *S. cerevisiae* Cdc14 binding partner in the nucleolus (Geil et al., 2008) and Tof2 is related to Net1 (Waples et al., 2009). A second *S. pombe* nucleolar protein that binds Clp1 is Dnt1 (Jin et al., 2007). *S. pombe* Dnt1 is related to both *S. cerevisiae* Net1 and Tof2 (Jin et al., 2007).

We first tested whether rDNA silencing was perturbed in *nsk1∆* cells because Dnt1 , Net1, and Tof2 are all involved in transcriptional silencing of rDNA (Geil et al., 2008; Huang et al., 2008; Jin et al., 2007; Straight et al., 1999). For this, we monitored for de-repression of the *ura4 ^+^* reporter gene that had been integrated into the normally silenced rDNA repeats (Jin et al., 2007; Thon and Verhein-Hansen, 2000). Depression of the *ura4 ^+^* reporter in this context leads to enhanced growth on plates lacking exogenous uracil and increased sensitivity to 5-fluoroorotic acid (5-FOA). By this assay, we found that rDNA silencing was partially relieved in *nsk1∆* cells ( Figure 1A ) and to the same extent as in *dnt1∆* cells (Jin et al., 2007). We next asked whether *nsk1∆* interacted genetically with *nuc1-632* , a mutant of the largest subunit of DNA-directed RNA polymerase 1, that is critical for the function and structural integrity of the nucleolus (Hirano et al., 1989). Although *dnt1∆* showed a negative genetic interaction with *nuc1-632* cells (Jin et al., 2007), we found no evidence of genetic interaction between *nsk1∆* and *nuc1-632* ( Figure 1B ). *nsk1∆* also did not have any genetic interaction with *dnt1∆* ( Figure 1C ).

We next examined whether other nucleolar proteins localized normally in *nsk1∆* cells because in the absence of Dnt1 or *S. cerevisiae* Net1, several nucleolar proteins are delocalized implicating Dnt1 and Net1 in nucleolar organization (Jin et al., 2007; Straight et al., 1999). Gar2-GFP, a nucleolar marker and ortholog of human nucleolin (Gulli et al., 1995; Rutherford et al., 2024), localized normally within the nucleolus of *nsk1∆* cells, as did two other nucleolar proteins, Nop2 , a rRNA methyltransferase involved in ribosome biogenesis (Rutherford et al., 2024), and Nuc1 (Hirano et al., 1989) ( Figure 1D ).

Tof2 is required for proper segregation of the rDNA during anaphase in *S. cerevisiae* (Geil et al., 2008). In *S. pombe* , the nucleolar DNA segregates after the bulk of the genomic DNA (Granot and Snyder, 1991; Strunnikov, 2005). Defects in rDNA structure lead to lagging rDNA during anaphase and asymmetries in rDNA segregation, easily visualized by monitoring Gar2-GFP (Win et al., 2005). Time-lapse imaging of 21 *nsk1 ^+^* and 42 *nsk1∆* cells showed that Gar2-GFP segregated equally and without lagging in all cells ( Figure 1E ).

We conclude from the combination of these experimental results that Nsk1 has no significant role in rDNA transcription, segregation of rDNA repeats during mitosis, or nucleolar organization but it does contribute to rDNA silencing during interphase. We speculate that this role may be related to its ill-defined function at centromeres during mitosis (Chen et al., 2011).

## Methods

*S. pombe* strains used in this study were grown in yeast extract (YE) or Edinburgh minimal media (EMM) plus selective supplements (Forsburg and Rhind, 2006). Strain construction was accomplished through tetrad analysis using standard methods (Moreno et al., 1991). Tagged strains were generated by endogenously tagging the 3′ end of open reading frames with sequences encoding *mNG:hphMX6, mCherry:natMX6, RFP:kanMX6,* or *GFP:kanMX6* using pFA6 cassettes, as previously described (Bahler et al., 1998) and lithium acetate transformations (Keeney and Boeke, 1994). G418 (100 mg/mL; Sigma-Aldrich), Hygromycin B (50 mg/mL; Thermo Fisher), or nourseothiricin (100 µg/ml; Sigma-Aldrich) was used for selection of *kanMX6, hphMX6,* or *natMX6* cells, respectively. Tagged strains were confirmed by whole-cell PCR. All fusion proteins were expressed from their native promoters at their chromosomal loci.

For rDNA silencing assays, cells were grown to log phase in YE at 32°C, a 10-fold serial dilution series starting with 50,000 cells were spotted on MAL (minimum media supplemented with 225 mg/L adenine and 75 mg/L leucine), YE, and YE containing 1.5 g/L of 5-Fluroorotic acid (5-FOA; Toronto Research Chemicals, North York, Ontario, Canada) agar plates.

Ethanol fixation and DAPI staining were performed as previously described (Roberts-Galbraith et al., 2009). Fixed-cell images in Figure 1D were acquired with a Zeiss Axio Observer inverted epifluorescence microscope with Zeiss 63X oil (1.46 NA) and captured using Zeiss ZEN 3.0 (Blue edition) software and Axiocam 503 monochrome camera (Zeiss). Exposure times ranged from 100 to 250 ms.

Live-cell time-lapse imaging in Figure 1E was performed on a Leica Thunder Imager system including a DMi8 inverted microscope, a 63X plan apo oil objective (1.40 NA), a Leica K8 sCMOS camera, standard excitation and emission filters and an LED light source. Images were captured using Leica Application Suite X (LAS X) software. A CellASIC ONIX microfluidics perfusion system (Millipore Sigma) was used, and cells were loaded into Y04C plates for 10 s at 8 psi. YE liquid medium flowed through the chamber at 5 psi throughout imaging. Z-series optical sections were taken at 0.5 µm spacing and images were acquired every 2 min.

## Reagents

***S. pombe* strains used in this study**

**Table:** 

**Strain**	**Genotype**	**Source**
972	* h ^-^*	Lab stock
KGY8393	* nsk1Δ::kanMX6 ura4-D18 ade6-M21X leu1-32 h ^-^*	This study
KGY4965	* dnt1Δ:: ura4 ^+^ ade6-M210 leu1-32 ura4-D18 h ^-^*	Jin et al., 2007
KGY7195	* nsk1Δ:: ura4 ^+^ dnt1 ::kanMX6 ura4-D18 ade6-M21X leu1-32 h ^+^*	This study
KGY8395	* Yip2.4pUC ura4 .7 nsk1Δ::kanMX6 leu1-32 ura4-D18 ade6-M21X h? *	This study
YDM2381	* Yip2.4pUC ura4 .7 ura4-DS/E leu1-32 ade6-216 h ^+^ ( ura4 ^+^ inserted at rDNA repeat) *	Dannel McCollum lab
KGY10029	*nop2-mNG:hphMX6* *nsk1Δ::kanMX6 ade6-M210 ura4-D18 leu1-32* h ^-^	This study
KGY10030-2	*nop2-mNG:hphMX6* *ade6-M210 ura4-D18 leu1-32* *h ^-^*	This study
KGY965-2	*nuc1-GFP* : *kanMX6* *sad1-mCherry:natMX6* aul? *h ^-^*	This study
KGY973-2	*nuc1-GFP* : *kanMX6* *nsk1Δ:: ura4 ^+^ sad1-mCherry:natMX6* aul? *h ^-^*	This study
KGY13130	* gar2-GFP:kanMX6 nsk1Δ:: ura4 ^+^ sid4-RFP:kanMX6 ade6-M210 leu1-32 ura4-D18 h? *	This study
KGY13131	*gar2-GFP:kanMX6 sid4-RFP:kanMX6 ade6-M210 leu1-32 ura4-D18 h?*	This study
KGY15219	*nuc1-632* *ade6-M210 leu1-32 ura4-D18 h ^-^*	Hirano et al., 1989
KGY15220	*nsk1Δ::kanMX6 nuc1-632* *ade6-M210 leu1-32 ura4-D18*	This stud
